# NeuroDNet - an open source platform for constructing and analyzing neurodegenerative disease networks

**DOI:** 10.1186/1471-2202-14-3

**Published:** 2013-01-03

**Authors:** Suhas V Vasaikar, Aditya K Padhi, Bhyravabhotla Jayaram, James Gomes

**Affiliations:** 1 Kusuma School of Biological Sciences, Indian Institute of Technology Delhi, Block 1A, Room No. 307, Hauz Khas, New Delhi 110016, India; 2 Department of Chemistry, Indian Institute of Technology Delhi, New Delhi, 110016, India; 3 Supercomputing Facility for Bioinformatics & Computational Biology, New Delhi, 110016, India

**Keywords:** Neurodegenerative diseases, Disease gene network, Protein-Protein interaction network, Boolean network, Systems analysis

## Abstract

**Background:**

Genetic networks control cellular functions. Aberrations in normal cellular function are caused by mutations in genes that disrupt the fine tuning of genetic networks and cause disease or disorder. However, the large number of signalling molecules, genes and proteins that constitute such networks, and the consequent complexity of interactions, has restrained progress in research elucidating disease mechanisms. Hence, carrying out a systematic analysis of how diseases alter the character of these networks is important. We illustrate this through our work on neurodegenerative disease networks. We created a database, NeuroDNet, which brings together relevant information about signalling molecules, genes and proteins, and their interactions, for constructing neurodegenerative disease networks.

**Description:**

NeuroDNet is a database with interactive tools that enables the creation of interaction networks for twelve neurodegenerative diseases under one portal for interrogation and analyses. It is the first of its kind, which enables the construction and analysis of neurodegenerative diseases through protein interaction networks, regulatory networks and Boolean networks. The database has a three-tier architecture - foundation, function and interface. The foundation tier contains the human genome data with 23857 protein-coding genes linked to more than 300 genes reported in clinical studies of neurodegenerative diseases. The database architecture was designed to retrieve neurodegenerative disease information seamlessly through the interface tier using specific functional information. Features of this database enable users to extract, analyze and display information related to a disease in many different ways.

**Conclusions:**

The application of NeuroDNet was illustrated using three case studies. Through these case studies, the construction and analyses of a PPI network for angiogenin protein in amyotrophic lateral sclerosis, a signal-gene-protein interaction network for presenilin protein in Alzheimer's disease and a Boolean network for a mammalian cell cycle was demonstrated. NeuroDNet is accessible at http://bioschool.iitd.ac.in/NeuroDNet/.

## Background

Neurodegenerative diseases (NDDs) are characterized by progressive neurological impairment caused by the accumulation of abnormal proteins and neuronal loss. The abnormal proteins apparently alter neuronal functions that lead to the disruption of synapses in neuronal sub-populations, neural circuitry and higher-order neural architectures within specific regions of the brain. Since neurological deficits are not necessarily associated with neuronal loss [1], it is very likely that neurodegenerative conditions might be caused by neuronal dysfunction. The challenge lies in understanding how aberrations in gene regulation, protein-protein interactions, and the consequent alterations in signaling and metabolic pathways results in neuronal dysfunction. The task is even more daunting considering the vast data that is being created by genomic and proteomic research in biological sciences. From the perspective of developing new therapies, important questions arise. How does one decipher signal-gene-protein interactions that drive neurodegeneration? How does one integrate interaction networks and clinical data to predict cognitive impairment and disease? To answer these questions, it will be necessary to rummage through information scattered across public-domain websites and research literature. Our objective is to gather and consolidate this information under one portal for NDDs and provide network tools to interrogate the data for identifying critical genes, determine pathways that are aberrant, create protein-protein interaction (PPI) networks to interpret disease mechanisms and perform qualitative and quantitative network analyses.

Previous instances of databases created to address specific problems underscores the importance of bringing together information under a common fold for analyses. The unifying principle for integrating this information was protein and gene interactions across species as in the case of BioGRID. BioGRID provides the interactions for *Saccharomyces cerevisiae*, *Schizosaccharomyces pombe*, *Caenorhabditis elegans*, *Drosophila melanogaster*, *Mus musculus* and *Homo sapiens*[2]. Each interaction record in BioGRID is based on experimental evidence and is linked to the supporting publication. MINT on the other hand contains the molecular interactions experimentally verified and reported in peer-reviewed journals [3]. Similarly, Reactome, a database of human pathways, was created with entries cross-referenced in a vocabulary associated with standard databases such as Uniprot, NCBI Entrez Gene, Ensembl, UCSC, HapMap, KEGG and primary research literature to PubMed. It describes the role of 5272 human proteins and 3504 macromolecular complexes in 3847 reactions organized into 1057 pathways [4]. Sage Bionetworks, ELIXIR, Biomart and InterMine, have recognized the value of collecting, curating and categorizing data, and have undertaken the colossal task of creating infrastructure for the management of open source databases [5-7]. Many other database resources available online have also been developed to unify a class of data of interest [8].

Data related to neurodegenerative diseases has also grown exponentially with recent advances in high throughput genotyping techniques using microarrays. To enable interpretation of the insurmountable data, databases have been created to gather and rationalize the impact of mutations and protein-protein interactions on clinical manifestation of individual diseases. Some of the examples include the databases for Alzheimer’s disease (AD), amyotrophic lateral sclerosis (ALS) and Parkinson’s disease (PD). The AD database AlzGene, was developed to understand the genetic proclivity of AD and predict candidates for other complex genetic diseases [9]. AlzGene catalogues all genetic association studies published in the field of AD. Meta-analyses results of polymorphisms with genotypes are publicly available at this site. Yang *et al.*[10] have floated a database that contains experimentally confirmed substantianigra expressed sequence tags from healthy and PD patients. The database captures genetic variation, differential gene expression, gene-regulating elements, mitochondrial proteins, and pathways associated with PD-related genes. To integrate genetic and clinical information on ALS, Yoshida *et al.*[11] developed a database that provides 180 unique variants identified in ALS patients along with the corresponding clinical data. These databases are useful to both experimentalists and theorists who wish to understand data pertaining to a single disease of their interest in relation to known information.

Many examples reported in the literature show that reconstruction and analyses of these networks has given a deeper understanding of disease mechanisms and strategies for therapeutic intervention. Goh *et al.*[12] has shown that NDDs possess one of the most connected networks through a disease-gene network analysis. Understanding these networks is important because genes associated with a disease are not randomly positioned, but occur in clusters that are positively correlated with other similar diseases [13]. The study of pathway-based genetic analysis in multiple sclerosis (MS) indicated that the understanding of biological mechanisms of disease pathogenesis and identification of drug targets may come from distant associations [14]. Hwang *et al.*[15] performed dynamic systems analyses to identify perturbations of cellular processes that were required for prion replication. PPI analysis of a network created by combining library and matrix yeast two-hybrid screens, led to the discovery that GIT1, a GTPase activating protein that modulates actin polymerization, spine morphology, and synapse formation in neuronal cells, enhanced the aggregation of the huntingtin protein. Further, through these analyses, they detected 6 new huntingtin interacting proteins of unknown function [16]. Limviphuvadh *et al.*[17] focused on protein–protein interaction networks associated with causative proteins of six neurodegenerative disorders. They investigated correlation among NDDs using domain characteristics and found that PD and HD showed highest correlation among them. However, the challenge lies in the development of a theoretical framework that will enable the organization of existing data, and permit the interrogation and interpretation of mechanisms causing disease.

It is in this light that we have created a database, NeuroDNet, that includes information about twelve neurodegenerative diseases - adrenomyeloneuropathy, Alzheimer disease, amyotrophic lateral sclerosis, ataxia-telangiectasia, dentatorubral-pallidoluysian atrophy, Friedreich ataxia, frontotemporal dementia, Huntington disease, Lewy body dementia, Parkinson disease, prion disease, progressive supranuclear palsy. It accounts for the interactions and regulation between signaling molecules, genes and proteins. This database is also the first of its kind, which enables the construction and analysis of NDDs through PPI, regulatory and Boolean networks. We also present the results of three case studies, which demonstrate the power of the analytical tools featured in NeuroDNet.

## Construction and content

### Database architecture

**Figure 1:**
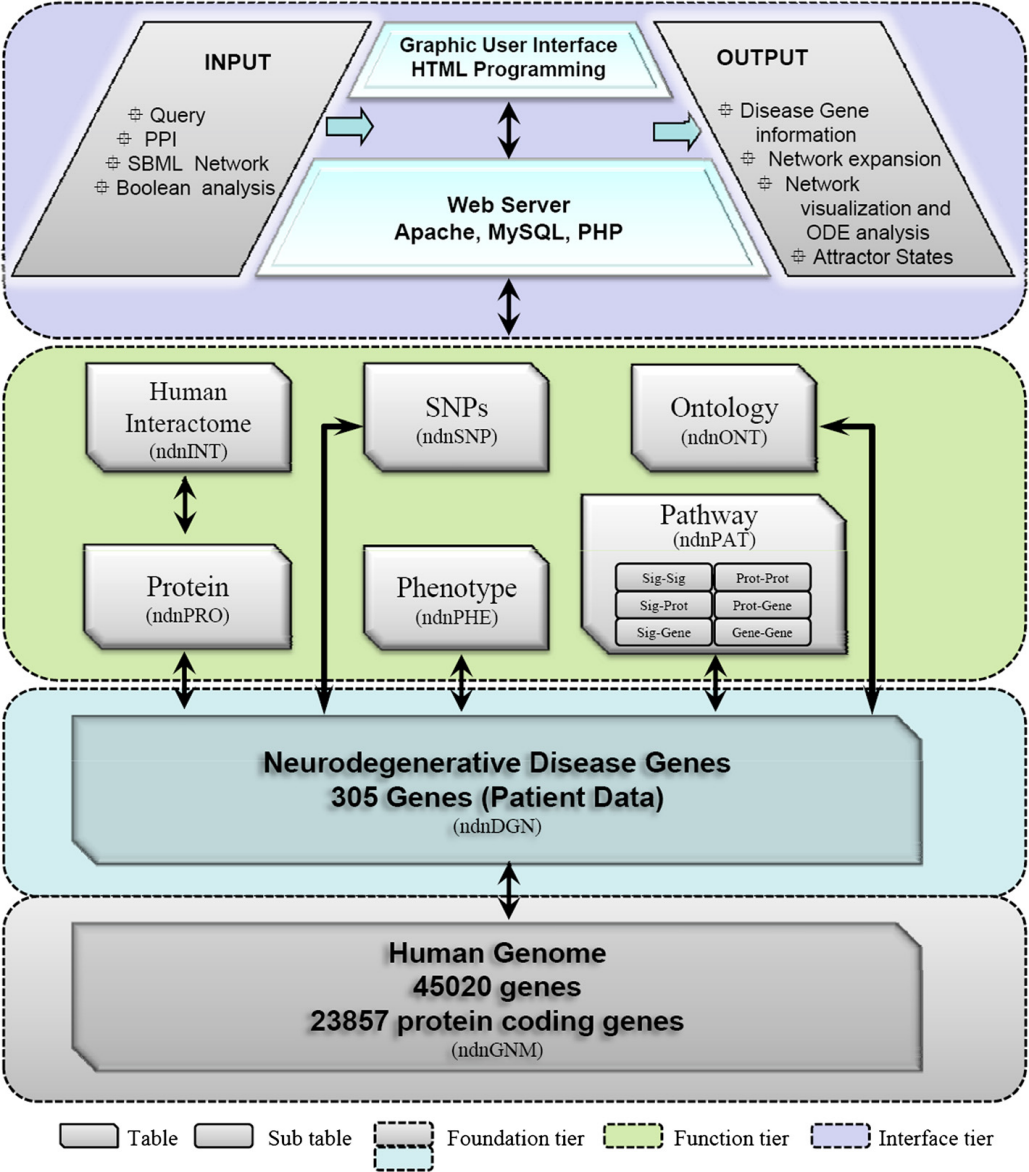
**NeuroDNet structure and organization.** The database has a three-tier structure - foundation, function and interface. The foundation tier consists of human genome data interlinked with the genes causing NDDs. The functional tier comprises proteins, human interactome, SNPs, gene ontology, phenotype and pathway tables associated with NDD-linked genes. The features offered by NeuroDNet are linked to these two tiers through a user interface.

The NeuroDNet database was developed in MySQL and is currently hosted on an APACHE http server located in the computer service centre of the Indian Institute of Technology Delhi. The database was built in a three-tier structure - foundation, function and interface (Figure 1). The foundation tier (ndnGNM) of NeuroDNet contains all the genes of the human genome-45020 genes of which 23857 are protein coding. The gene data for ndnGNM was acquired from the NCBI human genome database [ftp://ftp.ncbi.nih.gov/gene/DATA/GENE_INFO/Mammalia/]. Placed above ndnGNM is the table ndnDGN that contains genes associated with neurodegenerative diseases. Disease description and associated information was collected manually from MeSH [http://www.ncbi.nlm.nih.gov/mesh/] and OMIM [http://www.ncbi.nlm.nih.gov/omim/]. The 305 gene populating ndnDGN were the ones associated with patient data reported in the literature. These genes include those reported for sporadic and familial diseases, and in different ethnic groups.

The functional tier consists of six tables - ndnPRO (proteins), ndnINT (human interactome), ndnSNP (SNPs), ndnONT (gene ontology), ndnPHE (phenotype) and ndnPAT (pathway). These tables were populated with data obtained from various public databases. Since data is archived in different formats in these databases, the data acquired was recorded in a format that permitted seamless transition between different databases. The NCBI Gene ID was retained to enable a cross mapping of the NeuroDNet output to other databases. Data for the Protein table (ndnPRO) was obtained from Uniprot/Swiss-Prot database http://www.ebi.ac.uk/uniprot/. The protein-protein interaction data was assembled in two stages. First, the interaction data was downloaded from HPRD, BioGRID, DIP, MINT and Reactome [3,18,19]. Next, a PHP script was written to traverse across the downloaded files to capture the gene IDs, protein IDs and protein-protein interactions, and recorded in the ndnINT table. The data consists of 23857 genes possessing 51388 interaction edges. The SNP table was constructed using data obtained from the literature and OMIM database by screening only those that were associated with the NDDs. The gene ontology associated with the ndnDGN was acquired through a MATLAB script from the Gene Ontology Biological Process category and stored in the ndnONT table. Similarly, the phenotype associated with the disease genes was also obtained from published literature and from OMIM through NCBI; the retrieved data was manually verified and then stored in the ndnPHE table. Signaling pathway information obtained from images given in KEGG, Reactome, BIOCARTA, and Cell SnapShots was collated, converted into adjacency matrices and then stored in the ndnPAT table. The collected information was organized into six signal-protein-gene interaction sub-tables, namely, Sig-Sig (ndnPSS), Sig-Prot (ndnPSP), Sig-Gene (ndnPSG), Prot-Prot (ndnPPP), Prot-Gene (ndnPPG) and Gene-Gene (ndnPGG). The information contained in these six tables is used to create signal-protein-gene networks by NeuroDNet when a user accesses the “Network Model” feature of the interface programme. The network is constructed in SBML script assuming first order rates and output as an XML file which is ready for performing simulations in Celldesigner if the user can provide the kinetic parameters.

The user interface consists of two modules. The first module contains the algorithms coded in PHP. It performs computations required to complete the queries requested by the user. The second module, the graphical user interface communicates with the first module to execute a request. It is written in html and it provides the user with the features offered by NeuroDNet database.

### User interface

The home page of NeuroDNet gives a brief description of the database with a link for more information. There are four links given in the welcome panel - Query, Network, Disease Models and Boolean Analysis that enables a user to explore the different features of the website. The quick links are meant to take a revisiting user directly to a feature of interest. Using the first option “Query”, the database may be explored under the following categories - Disease, Gene, Protein, Polymorphism and Pathway. The class “Disease” contains the list of genes grouped by disease. For example, selecting “Alzheimer’s disease” gives the list of all the genes recorded in the database associated with AD. The user is free to pursue a gene of interest in this list by selecting links provided in the table displayed. “Gene ID” automatically connects the user to the corresponding gene page in NCBI, “Protein” to the corresponding Uniprot page for this protein, “Reference” to the research article, and “NDN Gene” to the NeuroDNet database page Gene Description. Gene Description comprises six sections - Gene, Protein, SNPs, Structures from PDB [http://www.rcsb.org/], PPI and Network Model. The database may be explored through links provided in each of these sections depending on the interest of the user. For example, selecting protein-protein interaction (PPI) option will display interacting proteins based on its cellular location. An option is provided to increase the neighbourhood degree for the protein. This enables the user to expand the PPI network to include related proteins in the analysis. At present, the upper limit on the degree is 3. We have also provided the user with the option of filtering the PPI network result by grouping it according to function, phenotype and occurrence in a pathway.

The second option “Network” contains three sub-categories - PPINet, DiseaseNet, and PathwayNet. The PPINet window accepts a list of genes or a text file that are part of a network under study. NeuroDNet processes this information and outputs a table where the connectivity of the network is shown in terms of the binary associations. When the simple example {A2M,APP,APOE} was submitted in the PPINet window, it gave the binary associations between A2M ↔ APOE and APOE ↔ APP. The output text file can be visualized graphically as a network using Cytoscape. The Entrez gene ID and Uniprot/Swiss-Prot protein ID given in the output text file are unique identifiers that may be used for global representation. The purpose of the “DiseaseNet” is to determine if interactions exist between the queried disease and other NDDs [12,20]. The result is displayed as a text file. “PathwayNet” lists pathways contained in ndnPAT that are associated with NDDs. The user may select any pathway of interest and determine the possible associations with other pathways in the NeuroDNet database. The pathways that possess crosstalk with the one of interest are given in a text file.

“Disease Model” is the third feature offered by NeuroDNet. It lists the collection of disease models that are linked to Celldesigner [http://www.celldesigner.org/] images and the corresponding SBML files stored in the database. These models were redrawn in Celldesigner from their original sources in reported literature. The user can download the SBML file from the database and visualize the model using a suitable network visualization tool. A PubMed reference is included with each model to refer the user to the original publication associated with it. These models may be easily expanded to include additional components for studying the dynamic behaviour of genes or proteins related to the disease.

The fourth option, “Boolean Analysis”, has been widely used to model regulatory networks and signaling pathways. This feature enables the user to determine the dynamic behaviour of a network of interest by assuming that each node of the network possesses a binary state. The Boolean analysis panel accepts two inputs, Nodes and Edges, for converting the network into matrices required for computation. An option is also available for specifying non-regulated nodes (self-degrading nodes). The user must provide the network information in the prescribed format through a window that accepts Boolean functions and select one of the two Boolean rules hosted by the database for determining the steady states of the network. Additional information and illustrative examples are given in the help link and user manual.

### Maintenance and update

All database entries were meticulously curated for accuracy. The following procedure was devised for data input, maintenance and update. The data was first acquired automatically by employing programme scripts. This primary information was checked against experimental findings described in the original source reported in the literature. Further, information about the associated pathways, phenotypes, proteins and their interactions were crosschecked and then annotated to the standard syntax. The error-free data was then stored as primary raw data files from which each of the six tables were updated automatically. The current data in NeuroDNet is up-to-date and will be updated quarterly.

## Utility and discussion

The database tools were created to facilitate disease analyses using a systems-based approach. Using these tools, the user can identify the critical genes or proteins associated with a NDD. The user may study the networks, for example, using graph-theoretic methods or study the dynamic behaviour of individual units or the complex assembly of units that constitute the network. Using NeuroDNet it is possible to evaluate hypothesis, query genes implicated or suspected in NDDs, examine experimental data in the light of gene and protein associations predicted by the network, visualize interactions and design experiments to validate predictions, and examine experimental data using information under one umbrella offered by this database. We present three case studies to illustrate the application of NeuroDNet.

### Case study 1: Protein neighbourhood network for human angiogenin protein

ALS is a debilitating neurological disease that affects humans across ethnic groups. Missense mutations in the protein angiogenin (ANG) that result in the partial or complete loss of angiogenic functions have been implicated with ALS [21,22]. Newer therapies for ALS may arise from a better understanding of mutations in ANG. Therefore, there is a need to understand how the loss of angiogenic properties may affect other proteins that are related directly or indirectly through various interactions at the cellular level. Through an exhaustive study of these interactions, it may be possible to elucidate how these mutations or SNPs lead to apoptosis of motor neurons.

Through the example of ANG, we illustrate how the database may be used to generate protein neighbourhood networks for visualization and analysis. ALS and ANG were selected from the Disease and Gene options from the Query panel respectively to enter the Gene Description page. The graphical view of PPI showed the first neighbourhood proteins {ACTC1, ACTN2, ATP6AP1, PTEN, RNH1, TDGF1, TNFSF8} and their locations in a cellular environment. The interactions may be visualized using the Cellular View or Graphical View; the result may also be downloaded and visualized in Cytoscape [http://www.cytoscape.org/] (Additional file 1: Figure S1).

**Figure 2:**
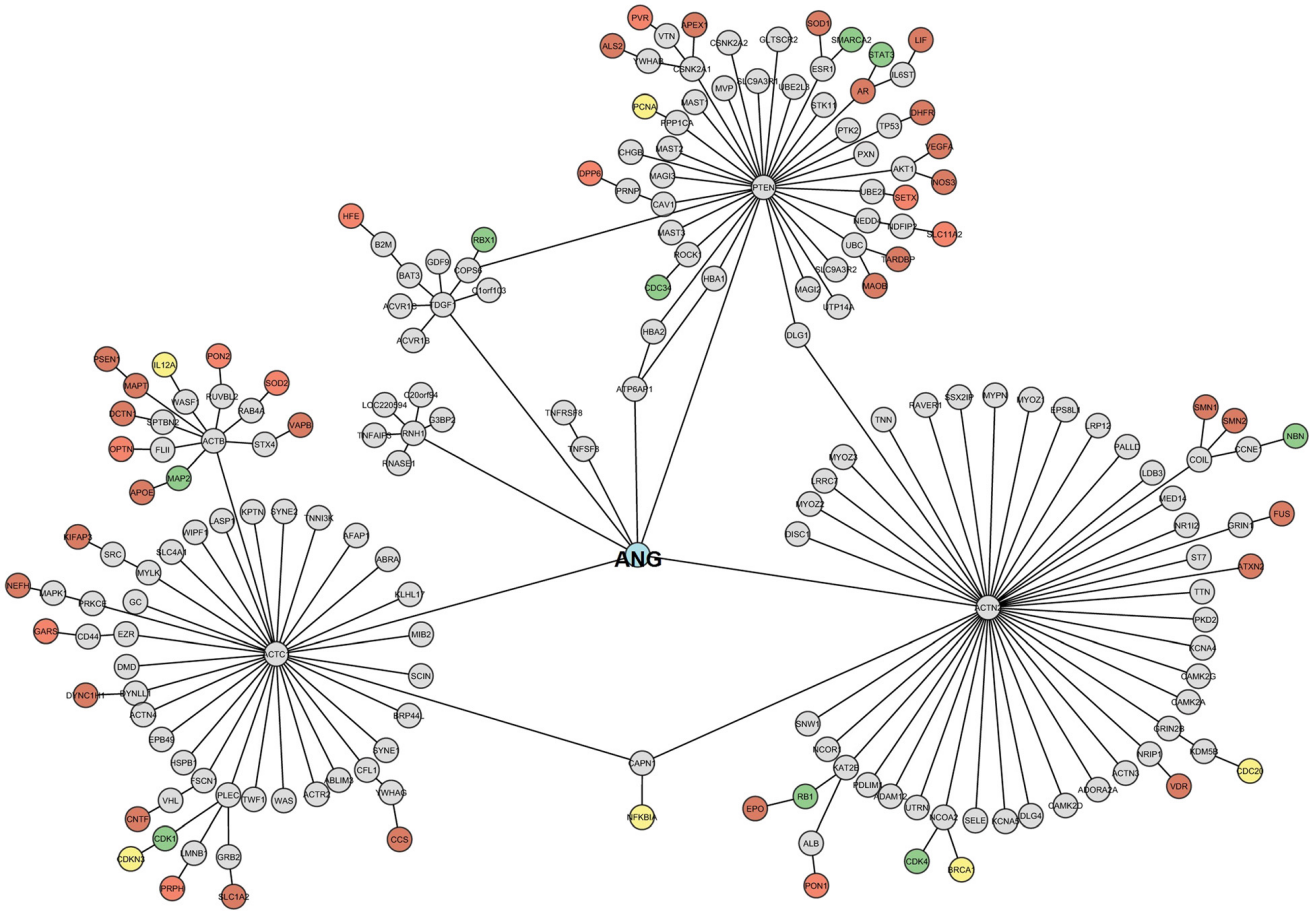
**PPI network showing how ANG and its neighbours are linked to genes causing ALS.** ANG (blue node) is connected to ALS associated proteins (red nodes) via intermediate proteins identified by expanding the network to include higher neighbourhood levels using NeuroDNet. Proteins that are up-regulated (green circles) or down-regulated (yellow circles) by ANG in HUVECs appear as the third neighbour of ANG.

Increasing the neighbourhood degree increases the number of interactions and complexity of the interaction network. ANG is an extracellular protein; when transported to the nucleus from the cytoplasm, it interacts with seven proteins (Additional file 1: Figure S1a) that participate in regulation of angiogenesis (RNH1), cardiac muscle development (ACTC1, TDGF1, PTEN), vasculature development (ANG, TDGF1, PTEN) and apoptosis (TDGF1, PTEN, ACTN2, TNFSF8, ATP6AP1). The second neighbours arising from these seven proteins include 119 proteins possessing 124 edges of interactions (Additional file 1: Figure S1b). Exploring the network further to include third neighbours recruited an additional 2580 proteins with 4424 edges of interactions (Additional file 1: Figure S1c). Network expansion to include higher degree neighbours is useful in identifying proteins engaging in crosstalk with other signalling pathways through direct or indirect interactions. Ahn *et al.*[23] had observed that ANG causes up regulation of Rb1, RBX1, NBN, CDK4, CDC34, SMARCA2, STAT3, CDK1, and MAp2; whereas down regulation of IκB-alpha, p55cdc, p35, BRCA, KAP, PCNA proteins in human umbilical vein endothelial cells (HUVECs). When the network was expanded to the third degree neighbour, we observed that all these proteins are included in the PPI network result. PPI networks, such as the one generated for ANG (Figure 2), may be created for other proteins and correlated with observed experimental observations.

### Case study 2: PSEN1 network model and effect on calcium homeostasis

Presenilin-1 and presenilin-2 (PSEN1 and PSEN2), are transmembrane protein modulators associated with early onset familial Alzheimer’s disease (FAD) [24]. It is a conserved polytopic transmembrane protein predominantly localised in endoplasmic/sarcoplasmic reticulum (ER/SR) and participates in the cleavage of the amyloid precursor protein (APP) which results in the formation of amyloid-β-peptides [25]. The aggregation of the “sticky” form, amyloid-β42, into fibrillar structures has been established as one of the mechanisms leading to all known clinical symptoms of Alzheimer’s disease. Consequently, we decided to investigate the role of PSEN1 in neuronal cell death in AD.

NeuroDNet tool under “Gene” was employed to construct the wiring diagram. This interactive tool allows the user to select a gene of interest from a disease category and retrieve interactions from the information contained in the six tables of “Pathway” to assemble the signal-gene-protein interactions. The information is retrieved and stored as an Excel spreadsheet, which is then converted into the SBML format using a PHP script. The XML file is displayed as a network using Celldesigner (Additional file 1: Figure S2).

PSEN1 is part of the γ-secretase complex involved in APP cleavage, notch signaling pathway and calcium homeostasis. The “Network Model” feature of NeuroDNet revealed that PSEN1 protein is an important vertex that regulates the cell proliferation, differentiation and apoptosis [26-28]. The network gives details of known interactions of PSEN1 mediated through the vertices Notch, APP and Ca^2+^. The information obtained may be used in verifying experimental results, building global systems model or examining specific phenomena. We illustrate how the interaction of the PSEN1 vertex with its neighbours may be used to understand how calcium leak from intracellular stores promotes neurodegeneration.

We asked the question whether the loss of channel function or its permanent opening caused by *PSEN1* mutation disrupted calcium homeostasis. To answer this question we first studied the PSEN1 interactions. The SBML network generated using NeuroDNet showed how Ca^2+^ interacts with channels and regulatory proteins. The differential equation model described by Marhl *et al.*[29] was obtained from the SBML repository http://www.ebi.ac.uk/biomodels/. This model explained complex intracellular Ca^2+^ oscillations using Ca^2+^ kinetics to describe fluxes from the ER and mitochondria to the cytoplasm, and the role of Ca^2+^ binding proteins in regulating the homeostasis.

**Figure 3:**
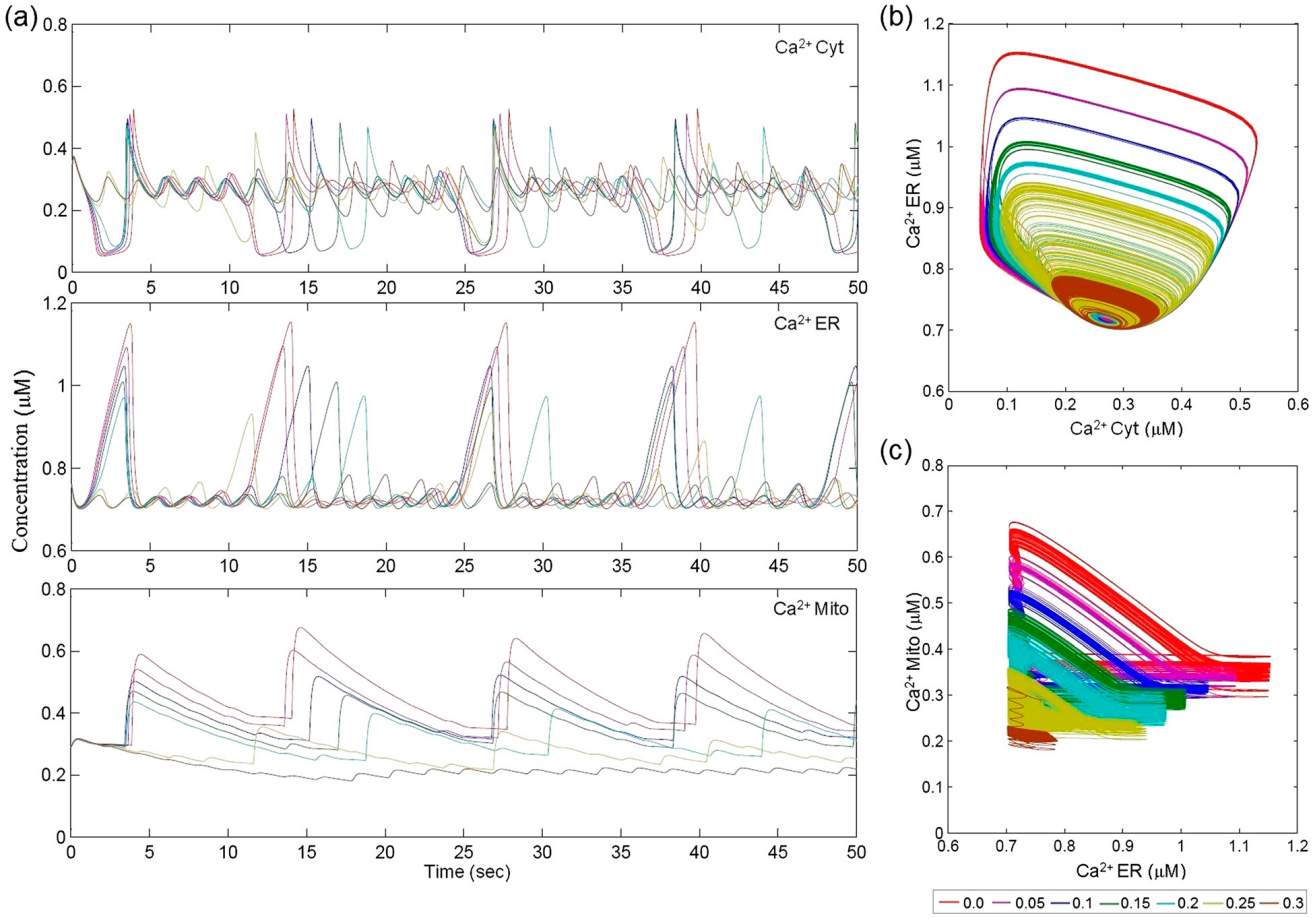
**Effect of leaky channel attributed to PSEN1 on calcium oscillations.** (**a**) Perturbation in rate constant *k*_*leak*_^*PSEN*1^, over 0.05-0.3 s^-1^ range affects amplitude and frequency of calcium oscillations in cytoplasm, mitochondria and ER. Absence of leaky current shows increase in ER and mitochondrial Ca^2+^ concentration (red line) whereas increase in leaky current decreases amplitude and increases frequency (brown line, 0.3 s^-1^) in cytoplasm. (**b**) Phase diagram between cytoplasmic and ER calcium store shows loss of regulation of calcium between two organelles. (**c**) Similarly, breakdown of ER-mitochondria Ca^2+^ control with increase in leaky current, is observed in ER-mitochondria phase diagram. The color scale for *k*_*leak*_^*PSEN*1^ is shown on the bottom right.

The calcium leak was attributed to the zymogen form of PSEN1 and the parameter associated with the corresponding equation, the rate constant *k*_*leak*_^*PSEN*1^, was perturbed (from normal to aberrant) over 0.05 - 0.3 s^-1^ range. We observed that the amplitude of cytoplasmic Ca^2+^ oscillations decreased while the frequency increased (Figure 3a). Absence of the “burst” signal of Ca^2+^ in the cytoplasm indicated a severe dysfunction. When the ER store of Ca^2+^ was depleted below 0.72 μM, the cytoplasmic control was lost completely (Figure 3b). Similar results were observed by Zampese *et al.*[30] in *PSEN2* mutant SH-SY5Y and HeLa cells. The mitochondrial regulation of the ER was also examined through a phase diagram. The limit cycle observed for normal parameter values disappeared when *k*_*leak*_^*PSEN*1^ increased to 0.3 s^-1^, suggesting breakdown of ER-mitochondria Ca^2+^ control (Figure 3c). Our results suggest that PSEN1 malfunction increased Ca^2+^ leak frequency, which in turn was responsible for the loss of ER-mitochondria Ca^2+^ regulation. Loss of this regulation effectively nullified the mitochondrial Ca^2+^ induced Ca^2+^ release (mCICR). It was shown that changes in spatiotemporal concentration of mCICR caused a permanent opening of the permeability transition pore (PTP) which ultimately triggered apoptosis [31-33].

An alternative explanation emerges when the ability of presenilins to form ion channels is considered. The zymogen form of presenilins localized in the ER/SR function as calcium channels and is responsible for the “leak” calcium currents involved in calcium homeostasis [34,35]. The mechanism of calcium leak into the cytosol has remained largely unexplained. Hofer *et al.*[36] observed a decrease in free calcium in the ER lumen in studies where the SERCA (sarco/endoplasmic reticulum calcium ATP-ases) was inhibited by the addition of thapsigargin. This leak remained unaffected even when the RyRs and IP3Rs were inhibited indicating that the basal leak rate of calcium was dependent on other unknown mechanisms. However, evidence that is more recent suggests that RyRs and IP3Rs may participate in the calcium leak under certain pathological conditions and in apoptotic phenotypes. Structural studies have shown that the cytoplasmic domain becomes dysfunctional creating a “channel only” behaviour [37]. Under normal physiological conditions, basal calcium leak has been attributed to natural ionophores, golgi, endosomes, and the potential difference between ER and cytoplasm [38]. Although the literature presents conflicting views on how the basal calcium leak is regulated, it is clear that calcium imbalance in the cytosol may push the cell into apoptotic state. Aberration in the PSEN1 network has been implicated in AD and its role in calcium homeostasis deserves further experimental investigation.

### Case study 3: Mammalian cell cycle - NeuroDNet Boolean analysis tool

Biological networks comprise the intricate circuitry of signals, genes, proteins and metabolites that brings about the kaleidoscope of changes within the cell. The interactions between the different molecular species, results in the observed complexity in cellular functions. Usually, computationally manageable sub-networks are constructed and analyzed to obtain insight into specific aspects of cellular behaviour. The dynamics of these sub-networks have been described with ordinary differential equations (ODE) but the ODE formalisms is constrained by the size of the sub-network and the dearth of kinetic and parametric information [39]. In such circumstances, it is more desirable to use Boolean networks, which are based on logical formalisms.

Boolean networks have been widely used to model gene regulatory networks and signaling pathways [40-42]. Each element of the system has a binary state ({0, 1} = {on, off}) and is therefore discrete, deterministic and parameter free [43]. The Boolean network is a graph, where the node (or vertex) *v* denotes in general, a gene or protein, and the edge *e* defines the nature of the interaction between two nodes. A network of *N* nodes has 2^*N*^ possible states. Time evolution of the network may be synchronous or asynchronous and the eventual steady states are called “attractor” states. The attractors, represented by the on-off state of genes in the network, correspond to important physiological states of the cells. For example, the state may indicate growth, differentiation or apoptosis [44]. Boolean analyses are particularly useful in cases where a qualitative insight is sought and for a better understanding of the network structure without invoking computationally intensive procedures.

More recently, the research has focused on developing generalized logical formalisms [45], examining robustness of networks [46], using interaction graph representations [47], investigating scalability across systems [48] and designing new efficient algorithms [49]. A number of systems such as, *A*. *thaliana* morphogenesis, yeast cell cycle, T-cell signaling and T-lymphocyte survival signaling, have been studied using Boolean networks to gain intuitive understanding [50-53].

**Figure 4:**
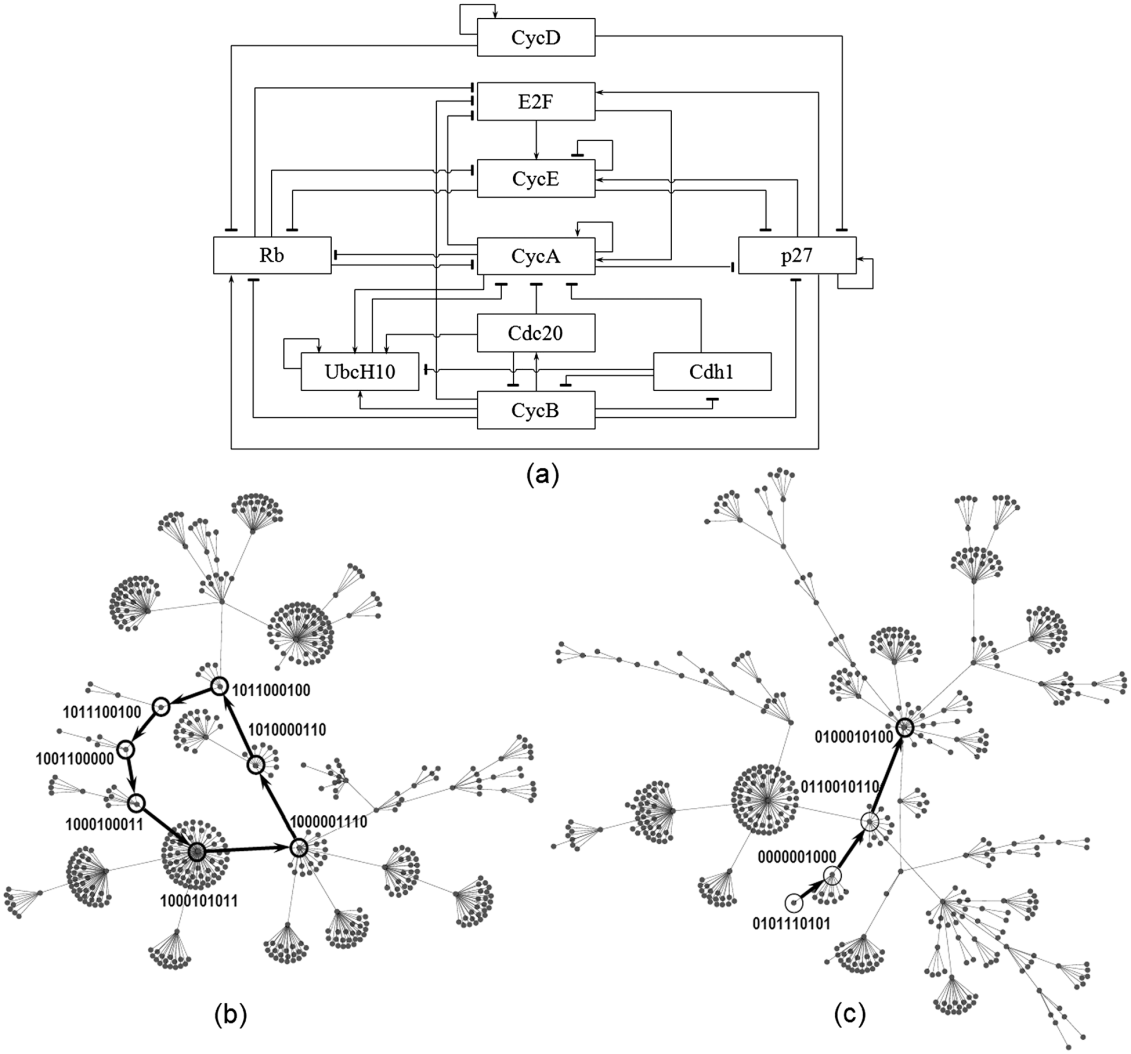
**Mammalian cell cycle network analysis using NeuroDNet.** (**a**) Cell cycle regulatory graph was redrawn from Fauré *et al.*[53]. Transition of states output of the NeuroDNet using a synchronous simulation has been shown. The cell cycle states through which a mammalian cell undergoes is shown (cyclic attractor) (**b**). The singleton attractor represents a steady state condition of the cell. The trajectory followed by the cell to reach G0 phase where intermediate nodes in the path depict the synchronous progression (**c**). The binary sequences shown in (**b**) and (**c**) correspond to the state of the nodes (*i* = 1, 2, …, 10) in the order given in Table 1 .

We illustrate the use of the Boolean analysis tool featured in NeuroDNet using the mammalian cell cycle described by Fauré *et al.*[54] (Figure 4a). Emerging evidence suggests that a strong relationship exists between the expression of cell cycle proteins and neurodegenerative diseases [55]. We describe the steps used to study this mammalian cell cycle to predict cellular fates (Figure 4a). The Boolean network comprising 10 nodes and 49 edges explained the emergence of G0 quiescent phase and non-spurious dynamical cycle. The Fauré cycle was reconstructed using NeuroDNet’s “Boolean Network” feature. The network properties {*G* = (10, 49)} were first keyed in. Next, the syntax for defining the logical operations of the network was written in the designated text window (Table 1). In the equations, the *i*^*th*^ node of a network was represented by *$nodei*, (*i* = 1, 2, …, 10). The Boolean rules and algorithm for computation were then selected and executed to obtain the attractor states (steady states). The sign associated with edge of the network, qualified the kind of interaction existing between two nodes - activation (+1) or inhibition (−1). At each node this information was processed using logic gates such as AND, OR and NOT. The dynamic status of each node was updated synchronously. The state of a node {0 or 1} was assigned the result of the logical operation at each instant, that is

(1) $$S_{i}\left(t+1\right)=\{\begin{array}{c} 1↔\sum_{j}a_{\mathrm{ij}}S_{j}\left(t\right)\geq 1\\ 0↔\sum_{j}a_{\mathrm{ij}}S_{j}\left(t\right)<1 \end{array}$$

**Table 1:** An example of the syntax for describing the logical operations of the mammalian cell cycle network

**Product**	**Node**	**Syntax**
CycD	$node[1]	$node[1] = $node[1];
Rb	$node[2]	$node[2] = (!($node[1]) & !($node[4]) & !($node[5]) & !($node[10])) | ($node[6] & !($node[1]) & !($node[10]));
E2F	$node[3]	$node[3] = (!($node[2]) & !($node[5]) & !($node[10])) | ($node[6] & !($node[2]) & !($node[10]));
CycE	$node[4]	$node[4] = ($node[3] & !($node[2]));
CycA	$node[5]	$node[5] = ($node[3] & !($node[2]) & !($node[7]) & !($node[8] & $node[9])) | ($node[5] & !($node[2]) & !($node[7]) & !($node[8] & $node[9]));
p27	$node[6]	$node[6] = (!($node[1]) & !($node[4]) & !($node[5]) & !($node[10])) |($node[6] & !($node[4] & $node[5]) & !($node[10]) & !($node[1]));
CDC20	$node[7]	$node[7] = $node[10];
CDH1	$node[8]	$node[8] = (!($node[5]) & !($node[10])) |$node[7] |($node[6] & !($node[10]));
UbcH10	$node[9]	$node[9] = !($node[8]) |($node[8] & $node[9] & ($node[7] | $node[5] | $node[10]));
CycB	$node[10]	$node[10] = (!($node[7]) & !($node[8]));

*!,&, | represents NOT, AND, OR logic gate.

Where *S*_*i*_(*t*) and *S*_*i*_(*t* + 1) represent the successive states of the *i*^*th*^ node, *S*_*j*_(*t*), *a*_*ij*_ = ±1 represents interacting nodes depending on whether the edge is activating or inhibiting. The network details submitted by the user are processed by an algorithm written in PHP script residing in the user interface module. The algorithm outputs a text file containing the 2^*N*^ Boolean outcomes and the list of singleton attractors and cyclic attractors which signify the states that the network can acquire. The output text files can be visualized for determining dynamic trajectories in a suitable network visualization tool such as Cytoscape (Figure 4b, c). Each attractor state represented as the binary sequence shown in (b) and (c), corresponds to the state of nodes taken in the order given in Table 1.

Cyclin D (CycD) controls the expression of retinoblastoma (Rb), a key tumor suppressor. During the G1 to S phase transition, E2F transcription factor (E2F) activates transcription of Cyclin E (CycE) and Cyclin A (CycA), which in turn controls the anaphase-promoting complex (APC) in cyclic fashion. We obtained one singleton attractor state that represented the quiescent G0 phase {0100010100} where each Boolean state corresponds to node (*i* = 1, 2, …, 10) (Table 1). The other stable states represent cyclic attractors consisting of seven successive states describing dynamical cycle consistent with those reported by Fauré *et al.*[54]. Mutational studies were also performed using this cell cycle network. The Boolean network for Rb mutant was executed and it was observed that the network lost its singleton attractor. Instead, a cyclic attractor was created that depicted lack of restriction point as shown by Novak and Tyson [56]. The results of this example showed that Boolean modeling tool correctly predicted the outcomes of the network and its dynamic behaviour qualitatively.

## Conclusion

NeuroDNet contains comprehensive information about the twelve neurodegenerative diseases under one portal. The database has a three-tier structure. Since the foundation tier contains data from the human genome upon which a table with disease-associated genes is constructed, it can be easily expanded by adding new tables containing genes of other diseases. NeuroDNet offers the user a bouquet of tools and features to analyze the information by creating PPI networks, signal-gene-protein interaction pathways and Boolean networks. The ANG case study shows how the PPI network may be extended to include higher degree neighbours and trace interaction routes between proteins of interest. The PSEN1 example demonstrated how the in-built programmes of NeuroDNet extracted the information contained in different tables of the database to create an interaction pathway. This case study also explained how PSEN1 is implicated in mCICR regulation. Finally, the Boolean analysis of the mammalian cell cycle was used to portray the power of qualitative analysis. In the absence of kinetic data, the Boolean network identified the physiological states of the cell cycle. The network generated by NeuroDNet predicted cell-cycle states and the G0 attractor state by a simple synchronous method. The features provided enable the user to identify unexpected disease linkages of genes and proteins. Higher degree neighbourhood networks created in NeuroDNet can be visualized to determine critical hubs and crosstalk associations between interacting partners. The tools may also be used to design directed experiments that provide better insight and reveal potential druggable targets. In future, NeuroDNet will be expanded to include all known neurodegenerative diseases.

## Availability and requirements

The current version of the NeuroDNet is hosted freely at http://bioschool.iitd.ac.in/NeuroDNet/.

## Competing interests

The authors declare that they have no competing interests.

## Authors’ contributions

The idea was conceived by JG and development carried out by SVV, JG, BJ. The clinical data collection, database organization and script writing was done by SVV. AKP helped in collection of ALS data. JG and SVV wrote the manuscript. All authors read and approved the final version of the manuscript.

## Supplementary Material

Additional file 1: Figure S1ANG neighbourhood network created using NeuroDNet. The complexity of ANG PPI network increases when higher degree neighbourhood interactions are considered. The figure shows ANG (yellow) and its first (a), second (b), and third (c) degree neighbours in light blue, dark blue and green, respectively. **Figure S2.** PSEN1 interaction pathway generated using NeuroDNet in SBML format and visualized using Celldesigner. PSEN1 is a part of γ-secretase complex involved in Notch signaling and APP processing. It also acts as Ca^2+^ leaky channel in ER (inset). The directed graph shows the interactions between the nodes [activation →; inhibition ]. The locations of nodes in cellular compartments are also shown. Here, the primary interaction of PSEN1 with γ-secretase and Ca^2+^ was used in NeuroDNet to create this extensive pathway that accounts for the main elements of Notch signaling, Calcium homeostasis, CAMKK cascade of events mediated through nodes like NOTCH1, NICD (notch intracellular domain) and CAM (calmodulin).Click here for file
