# Supplementary material for: NeuroDNet - an open source platform for constructing and analyzing neurodegenerative disease networks
Source: BMC Neurosci. 2013 Jan 3;14:3. doi: 10.1186/1471-2202-14-3 (PMC3570275; doi:10.1186/1471-2202-14-3)

**Additional file**

**Figure S1. ANG neighbourhood network created by NeuroDNet.** The complexity of ANG PPI network increases when higher degree neighbourhood interactions are considered. The figure shows ANG (yellow) and its first (a), second (b), and third (c) degree neighbours in sky blue, blue and green, respectively.


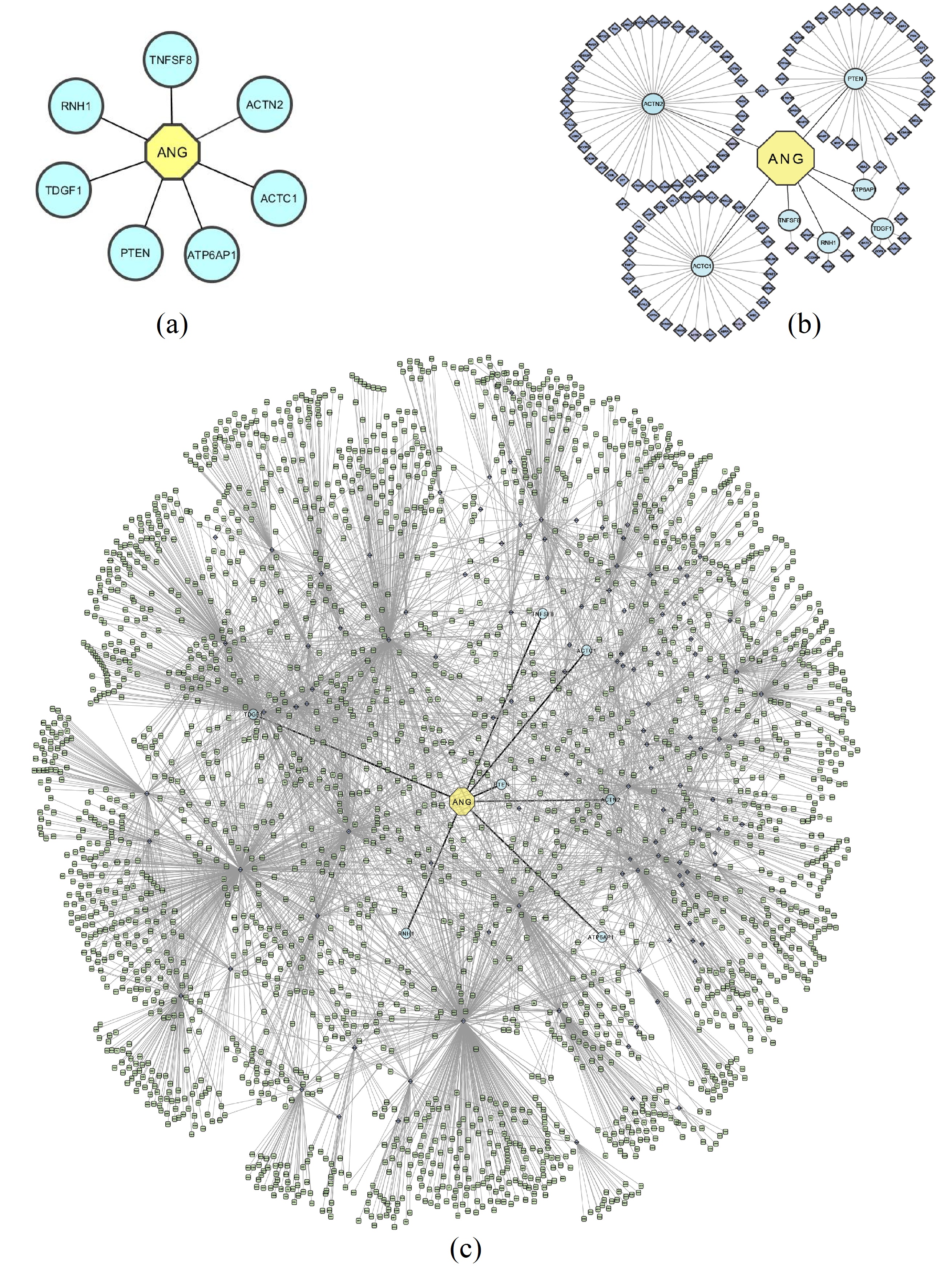


**Figure S2. PSEN1 interaction pathway generated by NeuroDNet in SBML format and visualized using Celldesigner.** PSEN1 is a part of γ-secretase complex involved in Notch signaling and APP processing. It also acts as Ca^2+^ leaky channel in ER (inset). The directed graph shows the interactions between the nodes [activation ; inhibition ]. The locations of nodes in cellular compartments are also shown. Here, the primary interaction of PSEN1 with γ-secretase and Ca^2+^ is used by NeuroDNet to create this extensive pathway that accounts for the main elements of Notch signaling, Calcium homeostasis, CAMKK cascade of events mediated through nodes like NOTCH1, NICD (notch intracellular domain) and CAM (calmodulin) can be visualized.


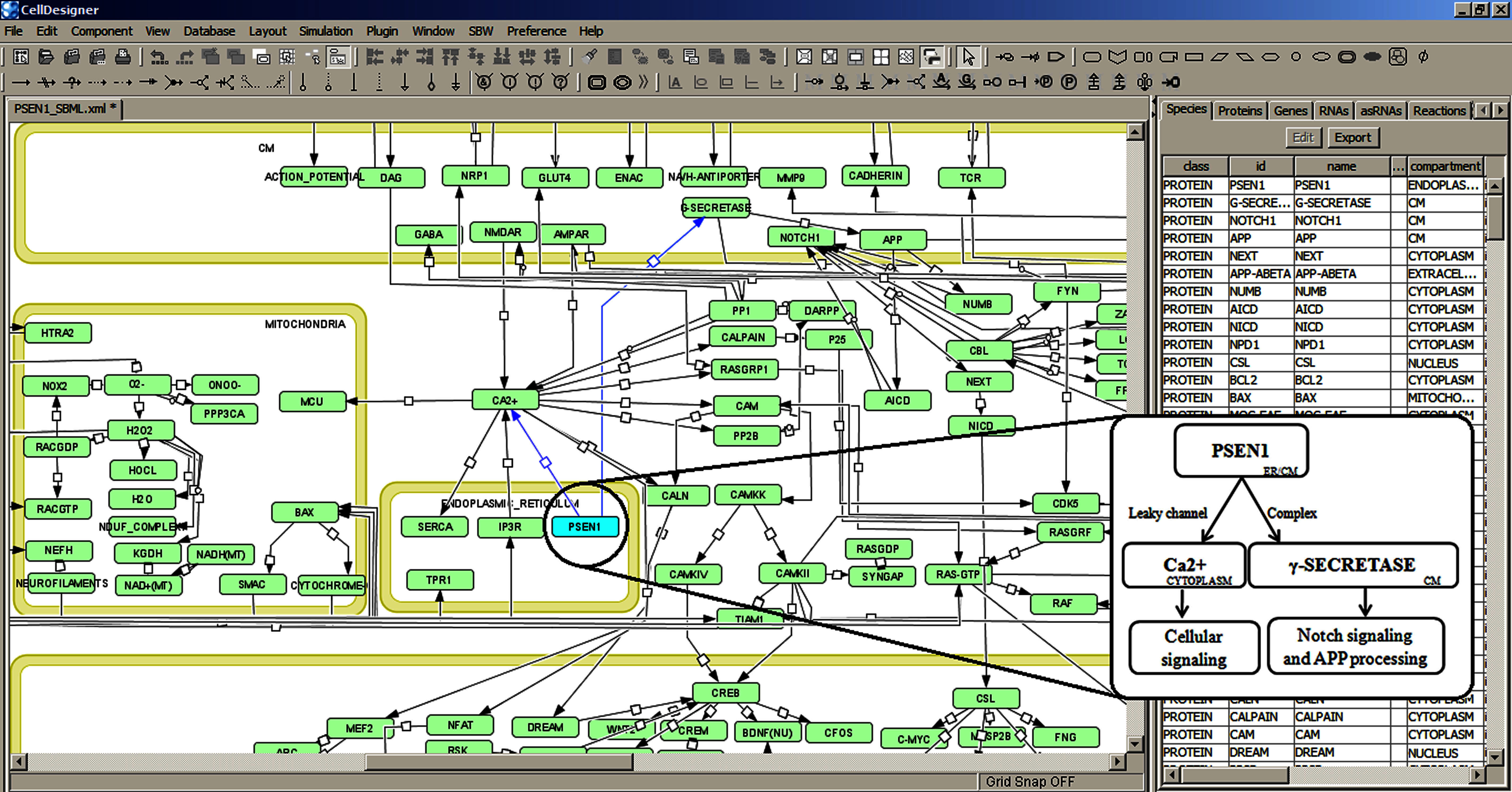

Supplement: Additional file 1: Figure S1 — ANG neighbourhood network created using NeuroDNet. The complexity of ANG PPI network increases when higher degree neighbourhood interactions are considered. The figure shows ANG (yellow) and its first (a), second (b), and third (c) degree neighbours in light blue, dark blue and green, respectively. Figure S2. PSEN1 interaction pathway generated using NeuroDNet in SBML format and visualized using Celldesigner. PSEN1 is a part of γ-secretase complex involved in Notch signaling and APP processing. It also acts as Ca2+ leaky channel in ER (inset). The directed graph shows the interactions between the nodes [activation →; inhibition ]. The locations of nodes in cellular compartments are also shown. Here, the primary interaction of PSEN1 with γ-secretase and Ca2+ was used in NeuroDNet to create this extensive pathway that accounts for the main elements of Notch signaling, Calcium homeostasis, CAMKK cascade of events mediated through nodes like NOTCH1, NICD (notch intracellular domain) and CAM (calmodulin). [file 1471-2202-14-3-S1.doc]
